# Comparative analysis of hemolymph proteome maps in diapausing and non-diapausing larvae of *Sesamia nonagrioides*

**DOI:** 10.1186/1477-5956-10-58

**Published:** 2012-09-28

**Authors:** Meritxell Pérez-Hedo, Isabel Sánchez-López, Matilde Eizaguirre

**Affiliations:** 1 Department of Crop and Forest Sciences, University of Lleida, AGROTECNIO Center, Rovira Roure 191, Lleida, 25198, Spain; 2 Proteomics and Genomics Scientific Technical Services, University of Lleida, Montserrat Roig 2, Lleida, 25008, Spain

**Keywords:** Diapause, Proteomic analysis, Hemolymph, 2D electrophoresis, Mass spectrometry, Sesamia nonagrioides

## Abstract

**Background:**

*Sesamia nonagrioides* is a noctuid that feeds on maize, sugar cane and sorghum in North Africa and Southern Europe. Larvae reared under long day conditions pupate after 5 or 6 larval instars, whereas larvae reared under short day conditions enter diapause and undergo up to 12 molts before dying or pupating. To better understand the mechanism of larval development and diapause, we identified proteins with different expressions in the sixth instar of diapausing and non-diapausing larvae.

**Results:**

A total of 52 differentially regulated proteins were detected in the hemolymph of the diapausing or non-diapausing larvae at the beginning or end of the sixth instar. From these proteins, 11 were identified by mass spectrometry (MALDI-TOF MS or MALDI-TOF/TOF MS/MS): 5 were upregulated in the hemolymph of non-diapausing larvae and 6 in the hemolymph of the diapausing larvae. Interestingly, some proteins were expressed only in non-diapausing larvae but none was expressed only in the hemolymph of diapausing larvae. The possible functions of some of these proteins related to diapause maintenance or to larval-pupal metamorphosis are discussed.

**Conclusions:**

The 2-DE proteomic map of *S. nonagrioides* hemolymph shows differential protein expression in diapausing and non-diapausing larvae. Some proteins that showed higher expression in the diapausing larvae at the end of the sixth instar could be involved in JH level maintenance thus in the diapause status maintenance. On the contrary, other proteins that showed the highest expression or that were expressed only in the non-diapausing larvae could be involved in larval-pupal metamorphosis.

## Background

The Mediterranean corn borer *Sesamia nonagrioides* (Lefèbvre) is a lepidopteran of the Noctuid species that feeds mainly on maize, sugar cane and sorghum. It is found in almost all Mediterranean countries, from the northern border at the 45th parallel to the southern border at the north of Africa [1,2], *S. nonagrioides* is a multivoltine species. In the area of this study, northeast Spain (Lleida), it has two complete generations and one incomplete third generation; the size of this third adult generation depends on the percentage of second-generation larvae that enter diapause [3]. The diapause is a hormonally regulated state with altered or reduced metabolic activity [4] determined genetically as a response to a series of stimuli that announce forthcoming adverse conditions for the insect [5]. A short-day photoperiod during the first and second instars induces larval diapause, the effect of the photoperiod being modulated by the temperature and the quality of the nutrients [6]. While non-diapausing larvae mostly molt to pupae after the sixth instar, diapausing larvae feed, move and molt with an indeterminate number of supernumerary molts [7,8]. Diapause in this species is related to increased levels of juvenile hormone (JH) in the hemolymph [9-12]. During diapause, the larvae maintain JH at a titer that allows retention of larval characters during the stationary molts that occur throughout diapause [13]. The sixth instar was chosen to carry out our study because it is the one in which the larva continues its normal development leading to pupation (non-diapausing) or remains as a larva, molting to another larval stage (diapausing).

The transcription of genes during larval diapause is crucial for understanding the molecular mechanism of this period [14-16]. During the diapause, several genes are downregulated, some are unique or upregulated, and some are related to stress proteins [17]. Recently, diapause in insects has been studied more and more at protein level [18-21], focusing on the perturbations of mRNA and protein abundance in the cells [22].

Proteomic knowledge of *S. nonagrioides* is still limited. In this study, we analyzed the effect of diapause induction on the hemolymph proteome of *S. nonagrioides* larvae. To this end, we compared the occurrence and expression of the hemolymph proteins between non-diapausing and diapausing larvae at two times of the sixth instar: a) two days after the molt to the sixth instar, when the pupation process in non-diapausing larvae has not yet begun; and b) seven days after the molt to the sixth instar, when the non-diapausing larvae are already starting the pupation process while the diapausing larvae maintain all the characteristics of the larval stage.

## Results

### Proteome of the hemolymph of *S. nonagrioides* larvae

The analyses to identify the differentially expressed hemolymph proteins were carried out on non-diapausing and diapausing larvae at the beginning (L6d2) and at the end (L6d7) of the sixth instar. The 2-DE gels were performed as indicated in the materials and methods section.

**Figure 1:**
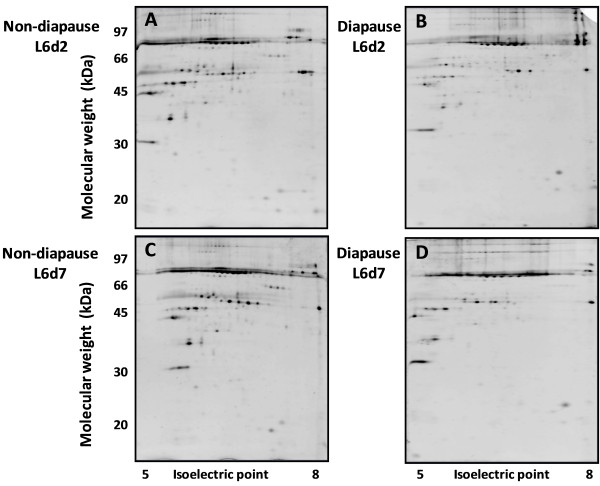
**Two-dimensional gel electrophoresis images of hemolymph protein extracts. A**: Proteome of L6d2 non-diapausing larvae; **B**: Proteome of L6d2 diapausing larvae; **C**: Proteome of L6d7 non-diapausing larvae; **D**: Proteome of L6d7 diapausing larvae.

The 2-DE analysis to identify differentially expressed hemolymph proteins is shown in Figure 1. The number of spots decreased as the larvae developed. At the beginning of the sixth instar (L6d2), 106 spots were detected in the hemolymph of diapausing and non-diapausing larvae, while at the end of the instar (L6d7), the numbers of spots detected was 98 in non-diapausing larvae and 87 in diapausing larvae.

### Differential 2-DE protein expression of *S. nonagrioides* hemolymph

**Figure 2:**
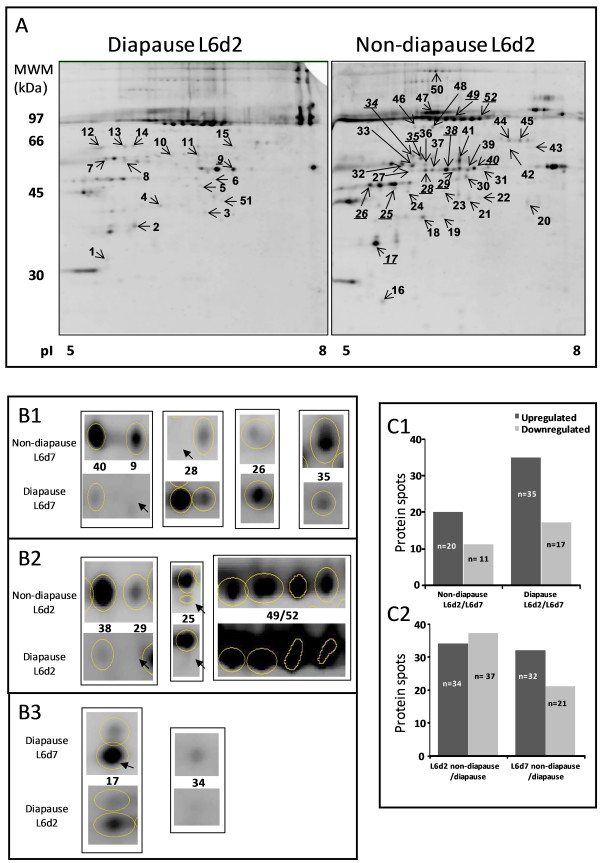
**Hemolymph proteome of *S. nonagrioides *larvae and its differential protein expression analysis. A**: Location of the differentially expressed proteins in the hemolymph proteome of L6d2 diapausing and non-diapausing larvae (selected as representatives); **B**: Close-up view of selected identified proteins from the total proteome; B1, protein expression of non-diapausing vs. diapausing larvae at L6d7; B2, protein expression of non-diapausing vs. diapausing larvae at L6d2; B3, protein expression at L6d7 vs. L6d2 in diapausing larvae. **C**: Number of qualitatively and quantitatively different protein spots; C1, L6d2 and L6d7 in the two photoperiod conditions; C2, non-diapausing and diapausing larvae at the two developmental points of the sixth instar.

Figure 2A shows the location of the differentially expressed proteins in the hemolymph proteome. The number of spots is allocated sequentially by the PDQuest analysis software, which analyzes in each case presence/absence or differential protein expression. A total of 52 non-redundant differential spots were derived from the four conditions of larval hemolymph studied: L6d2, L6d7, non-diapausing and diapausing.

The number of protein spots was lower at the end (L6d7) than at the beginning (L6d2) of the instar (Figure 2C.1). Hemolymph samples of non-diapausing L6d2 larvae showed 13 unique, 9 upregulated and 6 downregulated proteins compared with non-diapausing L6d7 larvae. Moreover, 5 unique spots were found in non-diapausing L6d7 larvae compared with non-diapausing L6d2 larvae. A similar result was found in diapausing larvae, in which L6d2 larvae contained 23 unique, 12 upregulated and 12 downregulated proteins compared with L6d7 larvae. Furthermore, 5 unique spots were detected in diapausing L6d7 proteome.

Figure 2C.2 shows the qualitative and quantitative differences between the proteomes of hemolymph of non-diapausing and diapausing larvae. In non-diapause L6d2 hemolymph 21 proteins were unique, 11 upregulated and 13 downregulated compared with diapause L6d2 hemolymph. In L6d2 diapausing larvae, 24 unique spots were also found. In L6d7, the hemolymph of non-diapausing larvae had 21 unique, 11 upregulated and 10 downregulated proteins compared with diapausing larvae. Also, 11 unique spots were found in L6d7 diapausing larvae.

### Identification of differential spots from the hemolymph proteome

**Figure 3:**
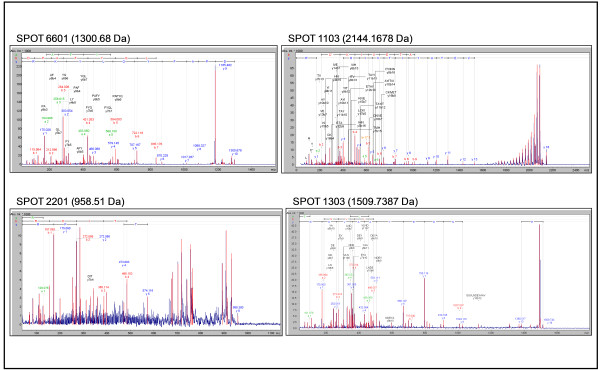
**Protein identification by MS/MS.** The MS/MS spectra of the proteins corresponding to spots 17, 26, 25 and 49–52 are shown. The spots were excised from preparative 12% polyacrylamide gels and digested with trypsin; the resulting peptides were analyzed by MALDI-TOF/TOF-MS/MS. The y and b ions and their corresponding sequences are shown.

Differentially expressed hemolymph proteins were identified by mass spectrometry, as described in the Materials and Methods section. The MS/MS spectra of some proteins are shown in Figure 3.

**Figure 4:**
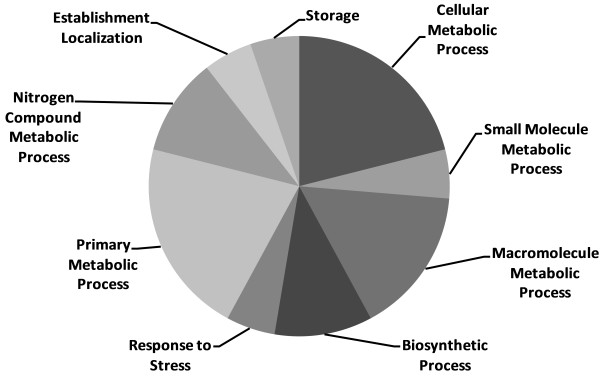
**Metabolic function of selected proteins identified in the hemolymph of *S. nonagrioides.*** The obtained protein sequences were functionally annotated using the Blast2GO tool.

Of a total of 85 differential spots detected from the gel analysis of the four conditions studied, (i.e. hemolymph from L6d2, L6d7, non-diapausing and diapausing larvae), the 52 non-redundant spots marked in Figure 2A were selected for identification. Of these, 11 spots were identified with confidence. Although PMF was performed for all spots, a statistical identification was obtained from only 7 of them (Table 1). The rest of the spots were analyzed by MS/MS and another 4 proteins were identified (Table 2). Figure 4 displays the sequence identification of the unique peptides of these four proteins identified by MS/MS spectrometry.

**Table 1:** ***S. nonagrioides *hemolymph proteins separated by 2-DGE and their identification by peptide mass fingerprint(PMF)**

**Spot No**^**a**^	**Protein name**	**SilkDBm entry**^**b**^	**NCBI entry**	**No. peptides matched/ totalpeptides**	**Coverage (%)**	**Score**	**pI/Mw**^**d**^	**Matched peaks**	**Corresponding sequence**
**40**	Hypothetical protein AaeL_AAEL004438 [*Aedes aegypti*]	BGIBMGA 009618	XP_001649137 GI:157106034	6/28	22	63	6.98 / 24099.61	861.43	TFRPSVR
								943.41	ALADGENVR
								1000.52 1099.55	VGYKLHER
								1156.62 1574.81	RALADGENVR
									MALKAYTFGR
									ALSAEKPAESESNDK
									
**28**	Fatty acid transport protein [*Bombyx mori*]	BGIBMGA 006185	NP_001127727 GI:197209926	7/21	14	60	8.78/ 77749.65	1038.49	TGDTFRWR IMTSVDMTGTFKMK NWSMPDIFHENVK FKATAAHYIGEMCR QNKYVPLGVEEYEK YILATPPSATDRQHK TAPRDFSALWCYVK
								1589.77	
								1616.78	
								1654.82	
								1695.77	
								1697.84	
								1713.83	
**34**	Soluble guanylate cyclase 89Da [*Harpegnathos saltator*]	BGIBMGA 006322	EFN86146 GI:307208935	5/11	21	69	9.78 / 39305.80	1037.53	MQTSSEARI
								1156.62	GSQGARSILLR
								1179.60	AETRCSSLEK
								1425.79	AHAASAASAALAVCR
								1482.78	EMLLNGWQHLSR
**38**	Hypothetical protein KGM_15508 [*Danaus plexippus*]	BGIBMGA 012907	EHJ79053 GI:357631584	8/41	29	66	8.52/ 40500.84	971.47	NMEENYR
									GCSLGHLTR
								1000.53 1099.57	KNMEENYR
									QLLPEEFGGPIVPVK
								1622.84	KPSKGMFASFTSSFK
									AFMHYIQNAHPCR
								1649.84	ETKDTIECYFTLR
									NRAVMDITQMVALPNK
								1660.82	
								1675.88	
								1832.77	
**29**	Protein kinase C1 [*Plutella xylostella*]	BGIBMGA 014131	ADU04569 GI:315319167	4/22	44	56	7.70 / 76360.82	862.49	KNVYLVK
								971.46	FMPRFFK
								1585.69 1607.64	FFKQPTFCSHCK
									ADEENNCDDGGGDLK
**35**	Hypothetical protein KGM_22030 [*Danaus plexippus*]	BGIBMGA 004876	EHJ68443 GI:357613332	5/14	13	60	6.06 / 39389.93	904.52	IQNFIDR
								1032.54	IQNFIDRK
								1060.62	RIQNFIDR
								1716.85	QKWPLVHGIAIYHR
								1928.91	IAYLQSVSPEYAQFWK
**9**	Diacylglycerol kinase eta [*Drosophila melanogaster*]		DGKH_DROME ^**e**^	16/45	10	55	6.40 / 211525.06	1274.67	NMLFYAKDEK
								1635.90	SIANCKWTTLASVGK
								2503.30	FNMHKQCQVAVMPLGTGNDLAR
								1660.83	WSIMVFEKAIPVPK
								2592.20	MSISTEQEAMLTGMVTSANHHLR
								1479.80	NLCDTVDDLVCR
								1574.87	DDEQLAVKCDILR
								1695.81	AIYNVVEHSEPGRPK
								1337.68	TYYMTREMDK
								943.43	DKPVEIDK
								2263.13	NLNSSMKPNTILTTSTSPTKK
									RHSSHAPGLAVR
								1287.63	HSSHAPGLAVREFDK
								1650.88	FLSSSPAASR
									QLLQKTCK
									QRQHSISIQR
									
								1022.53	
								1018.63 1252.71	

^**a**^ Spot numbering refers to the numbers in Figure 2.

^b^ Accession number refers to the closest match in the SilkDBm database.

^c^ Accession number refers to the closest match in the NCBI Blastp against nr.

^**d**^ pI/ Mw(Da) were calculated from amino acid sequence.

^**e**^ Accession number refers to the closest match in the Swiss-Prot database.

**Table 2:** ***S. nonagrioides *hemolymph proteins separated by2-DGE and their identification with MALDI-TOF/TOF –MS/MS**

**Spot no.**^**a**^	**Identified protein**	**SilkDBm entry**^**b**^	**NCBI entry**^**c**^	**pI/Mr**^**d**^	**Matched peptides**	**Peptide sequences MS2**	**Score**	**Source species**
**49-52**	Arylphorin	BGIBMGA009027	GI:356713490	5.90 / 82797.74	1300.68:421-430	DPAFYQLYKR	73	*Bombyx mori*
**17**	Juvenile hormone binding protein	EM_EST:FS939601 ^**e**^	AAA68242 GI:726332	5.40 / 26849.89	2144.1678 :212-229	TLCKIVETAYITVIHNIR	83	*Heliothis virescens*
**26**	Serine proteinase inhibitor-1B/C	EM_EST:ES583946 ^**e**^	ACR56865 GI:238816981	5.12 / 43134.57	1509.7387:65-78	EGSVLNDEYAAVSR	94	*Mamestra brassicae*
**25**	Heat shock protein 68	BGIBMGA 014536	AEI58997 GI:336454476	5.69 / 69324.64	958.51:249-256	RDITSNPR	17	*Bombyx mori*

^**a**^ Spot numbering refers to the numbers in Figure 2.

^b^ Accession number refers to the closest match in the SilkDBm database.

^c^ Accession number refers to the closest match in the NCBI Blastp against nr.

^d^ pI/ Mw(Da) were calculated from amino acid sequence.

^e^ Accession number refers to the closest match in the Invertebrates_EST database.

A close-up view of the identified differential spots is shown in Figure 2B. Among them, the protein kinase C1 was present only in non-diapausing L6d2 larvae, and heat shock protein 68 was only found in non-diapausing conditions on both developmental points of the sixth instar. Three of the eleven proteins were expressed at a higher level in non-diapausing larvae and in L6d2 diapausing larvae, whereas the expression of these proteins was very low and even undetectable in diapausing L6d7 larvae proteome (diacylglycerol kinase, hypothetical protein KGM_22030 and hypothetical protein AaeL_AAEL004438). The other five proteins identified (juvenile hormone binding protein, serine proteinase inhibitor, fatty acid transport protein, soluble guanylate cyclase 89 Da and hypothetical protein KGM_15508) were more abundant in non-diapausing L6d2 and diapausing L6d7 larvae, so the concentration decreased from 2 to 7 days in non-diapausing conditions and increased sharply from 2 to 7 days of L6 in diapausing conditions. Finally, arylphorin showed higher expression at the beginning of the sixth instar in both non-diapausing and diapausing larvae, but expression in all cases was higher than in diapausing larvae.

The identified proteins differentially expressed in the hemolymph of *S. nonagrioides* larvae are involved in various biological processes, as shown in Figure 4: establishment of localization (1 protein), storage (1 protein), the nitrogen compound metabolic process (2 proteins), the primary metabolic process (4 proteins), response to stress (1 protein), the cellular metabolic process (4 proteins), the small molecule metabolic process (1 protein), the macromolecule metabolic process (3 proteins), and the biosynthetic process (2 proteins). Moreover, the functions of proteins can be divided into various categories, as is reviewed in the discussion.

## Discussion

During the sixth larval instar, differences in hormone levels, proteins and enzymes cause diapausing *S. nonagrioides* larvae to have extra larval molts while non-diapausing larvae display metamorphosis, molting to pupae [10,23]. Hemolymph bathes the interior larval body and plays an extremely important role in the transport and storage of nutrients [24]. Therefore, changes in metabolism between non-diapausing and diapausing larvae of *S. nonagrioides* might be detected in the hemolymph. Although some proteomic studies of insect hemolymph have been performed for *Bombyx mori *[25,26], *Manduca sexta *[27] and a few other insects, this is the first study of the proteome of *S. nonagrioides* hemolymph. The aim of the study was to detect differential protein expression in the hemolymph of diapausing and non-diapausing *S. nonagrioides* larvae during the sixth instar that could be involved in diapause.

Fifty-two differentially regulated proteins were detected in the hemolymph proteome of diapausing and non-diapausing larvae at the beginning and end of the sixth instar. Among these, eleven proteins were identified. The fact that hemolymph proteome displays a high dynamic range difficult obtaining sufficient amount of protein for assessing the identity with confidence, even though 600 μg of protein were used per a preparative gel. Five proteins were upregulated in the hemolymph of non-diapausing larvae and six in the hemolymph of the diapausing larvae. Among the identified proteins, seven (protein Kinase C1, heat shock protein 68, diacylglycerol kinase, soluble guanylate cyclase 89 Da, hypothetical protein KGM_22030, hypothetical protein AaeL_AAEL004438 and hypothetical protein KGM_15508) were identified in the hemolymph of Lepidoptera for the first time.

Six proteins showed high expression at the end of the sixth instar of the diapausing larvae: fatty acid transport protein (FATP), soluble guanylate cyclase 89 Da, juvenile hormone binding protein, serine proteinase inhibitor, hypothetical protein KGM_15508, and arylphorin protein*.* FATP must be involved in the storage of energy for use in adverse conditions in diapause, because it is involved in lipid metabolism and facilitates the uptake of extracellular long-chain fatty acids [28]. Soluble guanylate cyclase 89 Da (Gyc-89 Da) is a heterodimeric enzyme that synthesizes cyclicguanosine monophosphate (cGMP). In *Drosophila melanogaster*, it has been demonstrated that synaptic activity of the Gyc-89 Da neurons is required for adult eclosion [29], but no relationship between the protein and insect diapause has been demonstrated to date. Juvenile hormone binding protein (JHBP) transports the hydrophobic JH in noncovalent complexes from its site of synthesis in the corpora allata to peripheral target tissues [30]. JHBP levels change significantly and are largely regulated by JH itself [31]. The levels of JH are higher in diapausing than in non-diapausing *S. nonagrioides* sixth-instar larvae [10-12], so the JHBP protein might play an important role during diapause by binding and transporting JH within the hemolymph, avoiding its degradation by unspecific esterase or hydrolase. Other proteins that could be involved in diapause are the serine proteinase inhibitors (serpin), irreversible inhibitors of serine proteases that regulate proteolytic activities in both physiological and pathological situations [32]. Chen et al. [33] showed that serine proteinases maintain the developmental status in the diapausing pupae of *Delia antique*, and that serine proteinase inhibitors participate in the coordination of various immune responses. Hypothetical proteins are proteins predicted only from nucleic acid sequences and protein sequences with an unknown function [34]. In relation to diapause, the hypothetical protein KGM_15508 was found by similarity in Lepidoptera (*Danaus plexippus*) but with an unknown function. Arylphorin is the most abundant hemolymph protein in the sixth instar of *S. nonagrioides*. Although expressed in diapausing and non-diapausing larvae, it is more abundant in diapausing larvae at the beginning and at the end of the sixth instar. The spots that appeared close to each other in the gel could be different isoforms of arylphorin. The main functions of this protein are oxygen transport and nutrient reservoir activity. It might have an important role as a reservoir or cellular defence mechanism during diapause, as has been observed in other Lepidoptera [35].

Five proteins showed the highest expression in non-diapausing larvae and could be involved in larval-pupal metamorphosis. The hypothetical protein AaeL_AAEL004438, with the suggested name GrpE protein homolog, is required for the translocation of transit peptide-containing proteins from the inner membrane into the mitochondrial matrix in an ATP-dependent manner [36] and promotes the progress of the Hsp70 reaction cycle [37]. In *S. nonagrioides* this protein could be associated with the preparation for larval-pupal metamorphosis. Diacylglycerol kinase (DGK) belongs to a family of enzymes that catalyzes the conversion of diacylglycerol to phosphatidic acid, using ATP as a source of phosphate. Rachinsky et al. [38] observed that increasing the concentration of diacylglycerol within the corpora allata cells favors metamorphosis by decreasing the biosynthesis of JH, which prevents metamorphosis [39]. The members of the family of hypothetical protein KGM_22030 are involved in biosynthesis of molybdenum cofactor (Moco), which is required for the activity of molybdoenzymes [40]. This protein has not previously been reported to be related to metamorphic processes. Interestingly, two proteins were only expressed in non-diapausing larvae: protein kinase C1 and a heat shock protein 68. Protein kinase C1 (PKC) belongs to a family of protein kinase enzymes that are involved in controlling the function of other proteins through the phosphorylation of hydroxyl groups of serine and threonine residues in many signal transduction cascades. PKC can suppress JH action by preventing nuclear proteins from binding to JH-responsive promoters [41]. Moreover, PKC is involved in the molting process of insects, as there is evidence that the receptor of activated C kinase 1 and PKC signal transduction cascade is implicated in the 20-hydroxyecdysone-induced expression of transcription factor CHR3, which is a gene involved in the initiation of the molting process [42]. Furthermore, Fu et al. [43] indicated that during the larval-pupal metamorphosis calponin activates PKC, thus facilitating the action of 20-hydroxyecdysone. In *Drosophila melanogaster* the activity of PKC was shown to be necessary to mediate 20-hydroxyecdysone-induced expression of 14 specific proteins [44]. Heat shock protein 68 is involved in the modulation of various stress responses [45]. The expression of this protein, which is involved in several physiological functions during diapause, is especially variable between species: it has been reported to be highly expressed in diapausing stages in *Ostrinia nubilalis* or *Manduca sexta *[46] but downregulated in *Lucilia sericata *[47] and *Omphisa fuscidentalis *[15], in the present study the protein was detected only in the hemolymph of the non-diapausing larvae.

## Conclusions

The 2-DE proteomic map of *S. nonagrioides* hemolymph shows for the first time differential protein expression in diapausing and non-diapausing larvae of the Mediterranean corn borer. Some proteins that showed higher expression in the diapausing larvae at the end of the sixth instar could be involved in JH level maintenance and thus in the diapauses status maintenance. On the contrary, other proteins that showed the highest expression or that are expressed only in the non-diapausing larvae could be involved in the larval-pupal metamorphosis. The role of these proteins on the larval-pupal metamorphosis or in the diapause maintenance has to be studied more deeply.

## Methods

### Insects and sample preparation

*S. nonagrioides* cultures were established from insects collected in central Catalonia and reared on semi-artificial diets at 25°C [48]. Neonate larvae were divided into two groups, submitted to long-day (LD: 16 h light, 8 h dark) or short-day (SD: 12 h light, 12 h dark) photoperiod conditions and checked periodically. Samples of hemolymph of LD and SD larvae of the sixth (L6) instar were collected on the second (L6d2) and seventh (L6d7) day after the molt by clipping off a proleg with microscissors. Each sample contained 100 μl of the pooled hemolymph from 8–15 larvae. The samples were centrifuged for 10 min at 14 000 rpm at 4°C, and dissolved in 500 μ l of the lysis solution 7 M urea, 2 M thiourea, 1% C7BzO detergent, 40 mM Trizma Base (Protein Extraction Reagent Type 4, Sigma C0356) in the presence of protease inhibitors (Protease Inhibitor Cocktail, Sigma P2714). Total protein content in the supernatant was determined by the method of Bradford [49] using BSA as standard.

### Two-dimensional gel electrophoresis (2-DE)

Total protein extracts were separated by 2D-PAGE gels: analytical gels contained 50 μg of total protein extracts. Three experimental replicates were performed for each sample. For preparative gels 300 μg and 600 μg of protein were applied. Samples were mixed with rehydration buffer (7 M urea, 2 M thiourea, 1% C7BzO detergent, 40 mM Trizma Base, 50 mM DTT , 1% IPG buffer pH 3–10, and 0.002% bromophenol blue) to a total volume of 200 μL. After testing several conditions, the following protocol for 2-DE gels was used: Isoelectric focusing (IEF) of passively rehydrated 11-cm IPG strips (pH 5–8) was performed in a Protean IEF Cell system (Bio-Rad) following the manufacturer’s instructions. IEF used a sequential gradient procedure of 500 V for 30 min, 1000 V for 1 h, and 6000 V until a total of 35000 VoltH. The current limit was 50 μA per IPG strip. After IEF separation, the gel strips were incubated for 15 min in the equilibration buffer (375 mM Tris base, 6 M urea, 20% glycerol, 2% SDS) containing 2% DTT, followed by 15 min in the same buffer containing 2.5% iodoacetamide instead of DTT. Two equilibrated 11-cm gel strips were loaded in each 12% polyacrylamide gel (22 cm x 20 cm x 1 mm) for the second-dimension separation in an Ettan DALTsix Electrophoresis Unit (GE Healthcare) in 0.25 M Tris–HCl pH 8.8, 1.92 M glycine and 1% w/v SDS electrophoresis buffer, and 8 mA/gel was applied overnight.

### Image analysis

For protein detection, the gels were stained with fluorescent Flamingo stain (Bio-Rad) and the images were acquired with a Versadoc MP 4000 system (Bio-Rad). The analysis of the gel images was performed using the PDQuest software (8.0.1; Bio-Rad). After automatic spot detection and gel matching, analysis was checked by visual inspection edited manually. The quantity of each spot was normalized using the LOESS local regression model.

The final recorded changes in protein levels were based on densitometric analysis of three different sets of each condition sample, and only those spots that were detectable on all gels of a sample set were considered for evaluation. The average intensities of resolved spots were compared using qualitative (present or absence) and quantitative (2.0-fold increase or 0.5-fold decrease ratios) functions within the PDQuest software. A list of differential spots was generated for identification.

### In-Gel digestion

Two preparative 2D gels, with 300 μg and 600 μg of protein respectively, were prepared as described in section above and visualized with Oriole Fluorescent Gel Stain (Bio-Rad). After differential analysis, selected spots were manually excised from each gel and digested in duplicate with trypsin, using 96-well perforated plates and a MultiScreen™ HTS Vacuum Manifold system (Millipore). Each small gel piece with protein was minced, washed twice with deionized water and dehydrated with 50% ethanol in 50 mM NH_4_HCO_3_ for 10 minutes and then with 100% ethanol for another 10 minutes. The gel piece was reduced with 10 mM DTT in 50 mM NH_4_HCO_3_ for 1 h at 56°C and alkylated with 55 mM iodoacetamide in 50 mM NH_4_HCO_3_ for 30 min at room temperature in the dark. It was then washed twice in 50 mM NH_4_HCO_3_ for 15 minutes and dehydrated with 5% acetonitrile (ACN) in 25 mM NH_4_HCO_3_ for 15 minutes, with 50% ACN in 25 mM NH_4_HCO_3_ for 15 minutes twice, and finally with 100% ACN for 10 minutes. After total evaporation of the ACN, 15 μl of 20 ng/μl trypsin in 25 mM NH_4_HCO_3_ was added and the gel piece was kept at 4°C for 45 minutes in order to rehydrate it completely with the trypsin solution. Then, the gel piece was covered with 25 mM NH_4_HCO_3_ and incubated at 37°C overnight. After digestion, the protein peptides were collected, evaporated in a SpeedVac (Savant) and resuspended in 5 μl of 70% ACN/0.1% trifluoroacetic acid (TFA). If necessary, the minced gel was washed three times with 0.25% TFA in 50% v/v acetonitrile and twice with 100% ACN to collect the remaining peptides.

### Protein identification by mass spectrometry

Preparative 2-DE gels were visualized by fluorescent staining with Oriole (Bio-Rad). One μl of tryptic peptide solution of each digested spot was applied on a MALDI plate, dried at room temperature and covered with 1 μl of saturated α-cyano-4-hydroxycinnamic acid prepared in 50% v/v ACN containing 0.1% TFA.

Protein identification was performed by peptide mass fingerprinting (PMF) or MS/MS mass spectrometry in an AutoflexSpeed MALDI-TOF/TOF mass spectrometer (Bruker Daltonics). Mass spectra (mode reflectron, MH+) were acquired by FlexControl version 3.0 software (Bruker Daltonics) recording in the range 800–4500 Da, and the MS/MS information was obtained in LIFT (laser-induced forward transfer) mode. MS spectra were externally calibrated using Peptide Calibration Standard II (Bruker Daltonics).

The peak lists obtained were compared against the Swiss-Prot and non-redundant NCBI protein databases, the invertebrates EST database of the NCBI, and 14623 sequences generated by the annotation of silkworm genomic sequences (SilkDB, http://silkworm.swu.edu.cn/silkdb/doc/download.html) using the MASCOT software package (version 2.3, Matrix Sciences, UK; http://www.matrixscience.com). MS and MS/MS combined spectra by BioTools version 3.1 software (Bruker Daltonics) were also used. The search parameters were set as monoisotopic peptide masses, carbamidomethylation of cysteine and oxidation of methionine as fixed and variable modifications, respectively, one trypsin missed cleavage and a maximum of ±100 ppm for PMF peptide tolerance and ±0.4 Da for MS/MS tolerance. The search results from the combined spectra with a statistically significant Mowse score (*p* < 0.05) were accepted. Protein identification obtained from the silkworm genomic sequences was achieved by NCBI protein-protein BLAST (Blastp) search of the SilkDB entry against non-redundant protein sequences. The obtained protein sequences were functionally annotated using the Blast2GO tool [50].

## Abbreviations

2-DE gel: Two- dimensional electrophoresis gel; JH: Juvenile hormone; L6d2: Larvae of the sixth instar two days old; L6d7: Larvae of the sixth instar seven days old; LD: Long day photoperiod; SD: Short day photoperiod; p*I*: Isoelectric point; MS/MS: Tandem mass spectrometry; FATP: Fatty acid transport protein; Gyc-89 Da: Soluble guanylate cyclase 89 Da; cGMP: Cyclicguanosine monophosphate; JHBP: Juvenile hormone binding protein; ATP: Adenosine triphosphate; hsp70: 70 kilodalton heat shock proteins; DGK: Diacylglycerol kinase; Moco: Molybdenum cofactor; PKC: Protein kinase C1; CHR3: *Caenorhabditis elegans* orphan nuclear hormone receptor; 2D-PAGE: Two- dimensional polyacrylamide gel electrophoresis; IEF: Isoelectric focusing; LOESS: New procedure in SAS/STAT software for performing local regression; MALDI-TOF/TOF: Matrix-assisted laser desorption/ionization time of flight; LIFT: Laser-induced forward transfer; NCBI: National center for biotechnology information; BLAST: Basic local alignment search tool.

## Competing interests

The authors declare that they have no competing interest.

## Authors’ contributions

MPH and ME conceived the idea of proteomics study, designed the work and performed the samples preparation /analysis. ISL carried out protein extraction, 2-DE, image acquisition and MS/MS analysis. MPH and ISL performed statistical analysis and interpretation of data. All authors read and approved the final manuscript.
